# Proteomic analysis of regenerating mouse liver following 50% partial hepatectomy

**DOI:** 10.1186/1477-5956-7-48

**Published:** 2009-12-29

**Authors:** Hongcui Cao, Jiong Yu, Wei Xu, Xiaofei Jia, Jinfeng Yang, Qiaoling Pan, Qiyi Zhang, Guoping Sheng, Jun Li, Xiaoping Pan, Yingjie Wang, Lanjuan Li

**Affiliations:** 1 State Key Laboratory for Diagnosis and Treatment of Infectious Diseases, 1st Affiliated Hospital, College of Medicine, Zhejiang University, 79 Qingchun Road, Hangzhou, 310003, PR China; 2 Department of Surgery, 1st Affiliated Hospital, College of Medicine, Zhejiang University, 79 Qingchun Road, Hangzhou 310003, PR China

## Abstract

**Background:**

Although 70% (or 2/3) partial hepatectomy (PH) is the most studied model for liver regeneration, the hepatic protein expression profile associated with lower volume liver resection (such as 50% PH) has not yet been reported. Therefore, the aim of this study was to determine the global protein expression profile of the regenerating mouse liver following 50% PH by differential proteomics, and thereby gaining some insights into the hepatic regeneration mechanism(s) under this milder but clinically more relevant condition.

**Results:**

Proteins from sham-operated mouse livers and livers regenerating for 24 h after 50% PH were separated by SDS-PAGE and analyzed by nanoUPLC-Q-Tof mass spectrometry. Compared to sham-operated group, there were totally 87 differentially expressed proteins (with 50 up-regulated and 37 down-regulated ones) identified in the regenerating mouse livers, most of which have not been previously related to liver regeneration. Remarkably, over 25 differentially expressed proteins were located at mitochondria. Several of the mitochondria-resident proteins which play important roles in citric acid cycle, oxidative phosphorylation and ATP production were found to be down-regulated, consistent with the recently-proposed model in which the reduction of ATP content in the remnant liver gives rise to early stress signals that contribute to the onset of liver regeneration. Pathway analysis revealed a central role of c-Myc in the regulation of liver regeneration.

**Conclusions:**

Our study provides novel evidence for mitochondria as a pivotal organelle that is connected to liver regeneration, and lays the foundation for further studies on key factors and pathways involved in liver regeneration following 50% PH, a condition frequently used for partial liver transplantation and conservative liver resection.

## Background

Among all organs, the liver is unique in its ability to repair itself after suffering loss of tissue mass from surgical resection or pathogenic and toxic factors [1]. This is mainly attributable to the quick re-entry of highly differentiated quiescent hepatocytes into the cell cycle in response to liver injury [2]. If the liver incurs lethal damage resulting from the resection of 65-70% of the liver, hepatocytes will immediately begin to grow to compensate for the lost cells. The remaining liver will keep regenerating until the original liver mass is restored about 2 weeks later. The liver stops growing when the liver mass reaches within 10% (±) of the original liver mass. Regeneration of liver tissue has a threshold of 30% partial hepatectomy (PH), below which, liver regeneration slows down [2], whereas an 80% PH leads to liver regeneration failure and a high mortality rate [3,4]. Liver regeneration can be observed in living human liver transplantation and split-liver transplantation (SLT) [5] which revealed that the donor's liver size doubled after 7 days, and the recipient's doubled between days 7-14 after surgery and is restored to the original size 60 days later.

Although 70% (or 2/3) PH is the most studied model of liver regeneration [1,6], resection of approximately half volume of the donor liver is more often with partial liver transplantation [7]. Moreover, resection must be more conservative in the presence of underlying liver diseases or in elderly patients (e.g., ≥70 years of age) [7]. Major (>50%) hepatectomy in the presence of cirrhosis or steatosis significantly increased morbidity [8]. With the presence of liver steatosis, 30% or more of the remnant liver should remain in order to maintain viability. Furthermore, studies have revealed an increased benefit of ischemic preconditioning in patients with hepatic steatosis who had lower resected liver volume (<50%) [9], and extensive resections are generally not recommended for patients with cirrhosis [10]. Hemihepatectomy (i.e., resection of the right or left hemiliver) has now been successfully and frequently used for surgical removal of liver-associate tumors and cancers [11-13]. However, the molecular mechanisms of liver regeneration and the protein expression profiles of livers with clinically-relevant lower volume resection have not yet been reported. In this study, 50% PH in mice was conducted to mimic the clinical conditions where approximately half of the liver is surgically removed, and the proteomic changes were analyzed during liver regeneration after PH. This work may lay the foundation for further studies on key factors and cell markers involved in liver regeneration.

## Results

### Histological examination of the regenerating liver

**Figure 1:**
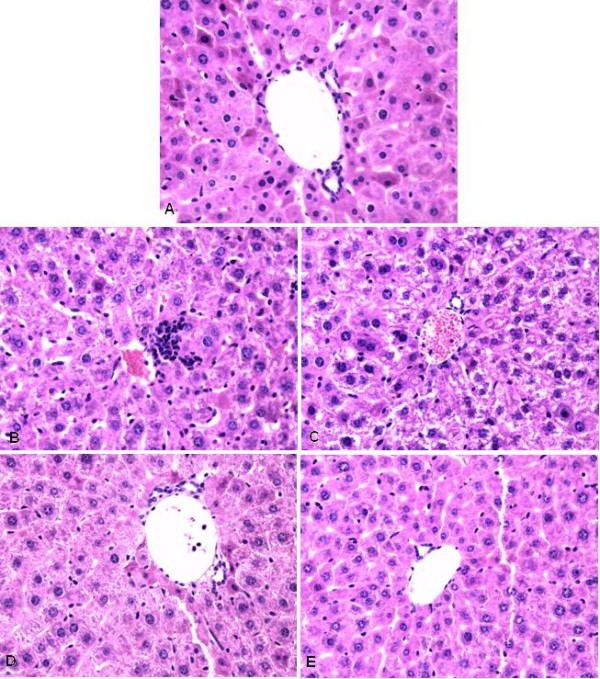
**Histological examination of regenerating mouse livers following 50% PH**. Fig.1(A) Liver histology slide of sham-operation group (HE staining 400×) showing clear hepatic lobule, radial liver cell cord, and clear hepatic sinusoid; Fig.1(B) Liver histology slide of 50% PH group 1 day after operation (HE staining 400×) showing cloudy swelling of some hepatocytes with focal piecemeal necrosis, and hepatocytes with multiple and enlarged nuclei started to appear. Fig.1(C) Liver histology slide of 50% PH group 3 days after operation (HE staining 400×) showing the majority of hepatocytes with double, multiple nuclei or single enlarged nuclei, prominent nucleoli, and cytoplasmic acidophilic bodies; Fig.1(D) Liver histology slide of 50% PH group 13 days after operation (HE staining 400×) showing double or enlarged nuclei but less prominent acidophilic bodies in most hepatocytes; Fig.1(E) Liver histology slide of 50% PH group 25 days after operation (HE staining 400×) showing double or enlarged nuclei still present in many hepatocytes which contain much reduced acidophilic bodies.

Staining with hematoxylin & eosin (H&E) revealed normal hepatic architecture within the clear hepatic lobule, radial liver cell cord, and clear hepatic sinusoid in no-surgery control and sham-operated mouse livers. In contrast, pathological symptoms were seen with livers subjected to 50% PH: 1 to 3 days post PH, liver cell acidophilic necrosis appeared and became intensified, and the majority of hepatocyte nuclei were increased in size, with vesicular bodies and prominent nucleoli caryocinesia, and stromal inflammatory cell infiltration; at 25 days, the degeneration of liver cells was lessened, but a substantial proportion of the hepatocytes still had enlarged nuclei indicative of cell division, which were seen even at 36 days (Fig. 1).

### Differentially expressed proteins in regenerating mouse livers following 50% PH

**Figure 2:**
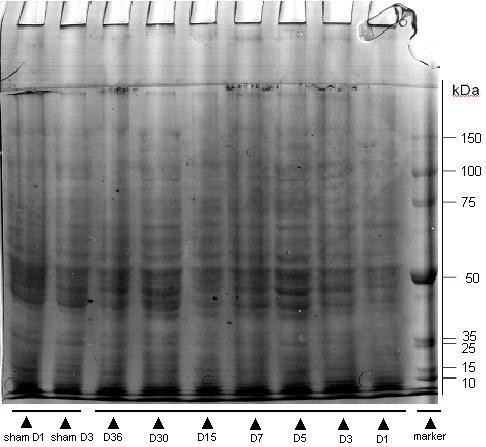
**Representative image of an SDS-PAGE gel separating mouse liver protein samples**. 60 μg of each sample from either sham-operated or 50% PH group recovered for different time period was separated on 10% SDS polyacrylamide gels. The gel was stained with Colloidal Coomassie Brilliant Blue G for 4 hours, and destained in a destaining solution.

Although we collected regenerating liver samples from day 1 through day 36 post 50% PH, data analysis has been focused on day 1 post PH as this is the time point when DNA and protein synthesis become most active [6,14] (Fig. 2). Among the 435 and 447 proteins identified respectively in the sham-operated and the 50% PH group, 87 proteins were found to be differentially expressed. They were categorized into four groups: group 1 in which 17 proteins were up-regulated with 50% PH (Table 1); group 2 in which 33 proteins were detected only in 50% PH group but not in sham-operated control, and thus are considered to be newly induced (Table 2); group 3 in which 15 proteins were down-regulated with 50% PH (Table 3); and group 4 in which 22 proteins were below detection limit in 50% PH group (i.e., detected only in sham-operated control but not in 50% PH group) (Table 4). In broad sense, proteins in group 1 and 2 (totally 50) can all be viewed as up-regulated proteins, and those in group 3 and 4 (totally 37) as down-regulated proteins in response to 50% PH. These differentially expressed proteins are involved in multiple critical metabolic and signaling pathways as classified in tables 1, 2, 3, 4.

**Table 1:** Up-regulated proteins in regenerating mouse livers 24 h post 50% PH (group 1)

IPI NUMBER	GENE SYMBOL	PROTEIN DISCRIPTION	SCORE	RATIO
**Carbohydrate, lipid and energy metabolism**				
IPI00111218	Aldh2	Aldehyde dehydrogenase, mitochondrial precursor	966.19	14.29
IPI00221890	Car3	Carbonic anhydrase 3	384.26	2.44
IPI00459487	Suclg2	Isoform 1 of Succinyl-CoA ligase [GDP-forming] beta-chain, mitochondrial precursor	446.22	1.75
IPI00226430	Acaa2	3-ketoacyl-CoA thiolase, mitochondrial	750.05	2.04
IPI00127558	Acox1	Acyl-coenzyme A oxidase 1, peroxisomal	199.01	3.85
**Cell regeneration-related proteins**				
IPI00885570	Actb	Beta-actin (Fragment)	200.18	1.19
**Amino acid and nucleic acid metabolism**				
IPI00134961	Acadm	Medium-chain specific acyl-CoA dehydrogenase, mitochondrial precursor	156.52	3.33
IPI00117914	Arg1	Arginase-1	399.59	5
IPI00134746	Ass1	Argininosuccinate synthase	237.27	3.45
IPI00111908	Cps1	Carbamoyl-phosphate synthase [ammonia], mitochondrial precursor	993.62	6.67
**Signal transmission**				
IPI00626662	Aldh1a1	Retinal dehydrogenase 1	1156.13	1.56
**Inflammatory factors and related proteins**				
IPI00165796	Gstm4	Glutathione transferase GSTM7-7	335.83	2.44
IPI00555023	Gstp1	Glutathione S-transferase P 1	807.98	1.64
IPI00283531	Gstp2	Glutathione S-transferase P 2	339.02	2
IPI00133903	Hspa9	Stress-70 protein, mitochondrial precursor	294.54	3.57
IPI00133522	P4hb	Protein disulfide-isomerase precursor	343.67	3.85
IPI00230108	Pdia3	Protein disulfide-isomerase A3 precursor	184.87	2

**Table 2:** Newly-induced proteins in regenerating mouse livers 24 h post 50% PH (group 2)

IPI NUMBER	GENE SYMBOL	PROTEIN DISCRIPTION	SCORE
**Carbohydrate, lipid and energy metabolism**			
IPI00466128	Akr1a4	Alcohol dehydrogenase	185.05
IPI00127206	Aldob	Fructose-bisphosphate aldolase B	275.21
IPI00469380	Aox3	Aldehyde oxidase 1	127.7
IPI00108939	Gapdhs	glyceraldehyde-3-phosphate dehydrogenase, spermatogenic	194.98
IPI00319994	Ldha	L-lactate dehydrogenase A chain	227.86
IPI00121079	Cyb5r3	Isoform 1 of NADH-cytochrome b5 reductase 3	128.55
IPI00130804	Ech1	Delta(3,5)-Delta(2,4)-dienoyl-CoA isomerase, mitochondrial precursor	333.62
IPI00469195	Echdc2	Isoform 1 of Enoyl-CoA hydratase domain-containing protein 2, mitochondrial precursor	94.22
**Cell regeneration-related proteins**			
IPI00461514	Hist1h2be	Hist_1_h_2_bc Histone H_2_B	122.68
IPI00187543	Hist2h2be	Histone H_2_B type 2-E	122.68
IPI00229539	Hist3h2bb	histone cluster 3, H_2_bb	122.68
IPI00348270	Hist2h2bb	Histone H_2_B type 2-B	122.68
IPI00648991	Hist1h2bp	Isoform 1 of Histone H_2_B type 1-P	122.68
IPI00129526	Hsp90b1	Endoplasmin precursor	305.35
IPI00406377	Krt7	Keratin, type II cytoskeletal 7	138.69
IPI00221797	Krt75	Keratin, type II cytoskeletal 75	260.86
IPI00785403	Krt6b	keratin complex 2, basic, gene 6b	260.86
IPI00131368	Krt6a	Keratin, type II cytoskeletal 6A	260.86
IPI00331459	Krt85	Keratin type II cuticular Hb5	139.91
IPI00139301	Krt5	Keratin, type II cytoskeletal 5	260.86
IPI00626239	Rpl11	Ribosomal protein L11	126
IPI00125521	Rps5	40S ribosomal protein S5	132.84
IPI00274407	Tufm	Isoform 1 of Elongation factor Tu, mitochondrial precursor	245.14
**Amino acid and nucleic acid metabolism**			
IPI00269076	Ak2	Adenylate kinase isoenzyme 2, mitochondrial	178.47
IPI00116222	Hibadh	3-hydroxyisobutyrate dehydrogenase, mitochondrial precursor	309.66
IPI00116603	Otc	Ornithine carbamoyltransferase, mitochondrial precursor	340.46
IPI00129178	Oat	Ornithine aminotransferase, mitochondrial precursor	166.18
**Signal transmission**			
IPI00317309	Anxa5	Annexin A5	168.8
IPI00230540	Vdac1	Isoform Mt-VDAC1 of Voltage-dependent anion-selective channel protein 1	139.28
**Inflammatory factors and related proteins**			
IPI00316509	Ephx1	Epoxide hydrolase 1	203.74
IPI00230185	Gpd1	Glycerol-3-phosphate dehydrogenase [NAD^+^], cytoplasmic	141.79
IPI00331322	Mgst1	Microsomal glutathione S-transferase 1	183.83
IPI00134131	Scp2	Isoform SCPx of Non-specific lipid-transfer protein	203.84

**Table 3:** Down-regulated proteins in regenerating mouse livers 24 h post 50% PH (group 3)

IPI NUMBER	GENE SYMBOL	PROTEIN DISCRIPTION	SCORE	RATIO
**Carbohydrate, lipid and energy metabolism**				
IPI00323592	Mdh2	Malate dehydrogenase, mitochondrial precursor	469.97	1.6
IPI00130280	Atp5a1	ATP synthase subunit alpha, mitochondrial precursor	777.7	1.42
IPI00230507	Atp5h	ATP synthase subunit d, mitochondrial	158.83	1.51
IPI00755311	Cat	Catalase	150.19	2.66
**Cell regeneration-related proteins**				
IPI00136929	Actg1	Gamma actin-like protein	440.24	1.46
IPI00129028	EG434428	similar to Tubulin, alpha 3c isoform 1	405.05	1.42
IPI00133456	Rgn	Regucalcin	385.17	2.77
**Amino acid and nucleic acid metabolism**				
IPI00130950	Bhmt	Betaine--homocysteine S-methyltransferase 1	526.33	1.42
IPI00117312	Got2	Aspartate aminotransferase, mitochondrial precursor	363.02	1.28
IPI00554931	Hpd	4-hydroxyphenylpyruvate dioxygenase	260.38	1.84
**Signal transmission**				
IPI00762128	Tst	33 kDa protein	192.75	3
**Inflammatory factors and related proteins**				
IPI00230212	Gstm1	Glutathione S-transferase Mu 1	1479.46	1.49
IPI00648333	Gstm4	Glutathione S-transferase, mu 4	339.86	1.95
IPI00323357	Hspa8	Heat shock cognate 71 kDa protein	151.26	1.58
IPI00319652	Gpx1	Glutathione peroxidase 1	184.93	1.55

**Table 4:** Proteins below detection limit in regenerating mouse livers 24 h post 50% PH (group 4)

IPI NUMBER	GENE SYMBOL	PROTEIN DISCRIPTION	SCORE
**Carbohydrate, lipid and energy metabolism**			
IPI00126625	Acsm1	Isoform 1 of Acyl-coenzyme A synthetase ACSM1, mitochondrial precursor	246.24
IPI00338536	Sdhb	Succinate dehydrogenase [ubiquinone] iron-sulfur subunit, mitochondrial precursor	141.26
**Cell regeneration-related proteins**			
IPI00119667	Eef1a2	Elongation factor 1-alpha 2	147.8
IPI00108125	Eif5a	Eukaryotic translation initiation factor 5A-1	107.8
IPI00121309	Ndufs3	NADH dehydrogenase [ubiquinone] iron-sulfur protein 3, mitochondrial precursor	114.89
IPI00762051	Rpl24	60S ribosomal protein L24	108.35
IPI00112448	Rps10	40S ribosomal protein S10	115.27
**Amino acid and nucleic acid metabolism**			
IPI00461964	Aldh6a1	Methylmalonate-semialdehyde dehydrogenase [acylating], mitochondrial precursor	302.15
IPI00120123	Dmgdh	Dimethylglycine dehydrogenase, mitochondrial precursor	147.51
IPI00114840	Endog	Endonuclease G, mitochondrial precursor	87.98
IPI00742399	Gamt	Isoform 1 of Guanidinoacetate N-methyltransferase	109.42
IPI00856685	Fah	16 kDa protein	176.55
IPI00856409	Got1	Protein	121.03
IPI00880958	Hpd	18 kDa protein	211.4
IPI00173179	Mettl7b	Methyltransferase-like protein 7B precursor	116.73
**Signal transmission**			
IPI00153317	Aldh1l1	10-formyltetrahydrofolate dehydrogenase	413.41
IPI00170307	Apoa1bp	Apolipoprotein A-I-binding protein precursor	127.86
IPI00134432	Ugt	Ugt1a6b;Ugt1a5;Ugt1a1;Ugt1a10;Ugt1a9;Ugt1a2; LOC632297;Ugt1a7c;Ugt1a6a UDP-glucuronosyltransferase 1-6 precursor	164.75
IPI00226356	Ttpa	Alpha-tocopherol transfer protein	171.27
**Inflammatory factors and related proteins**			
IPI00458306	Gstm2	Glutathione transferase (EC 2.5.1.18) class mu chain Yb2 (Fragment)	149.31
IPI00881469	Gstt1	glutathione S-transferase, theta 1	91.43
IPI00554929	Hsp90ab1	Heat shock protein HSP 90-beta	358.82

**Figure 3:**
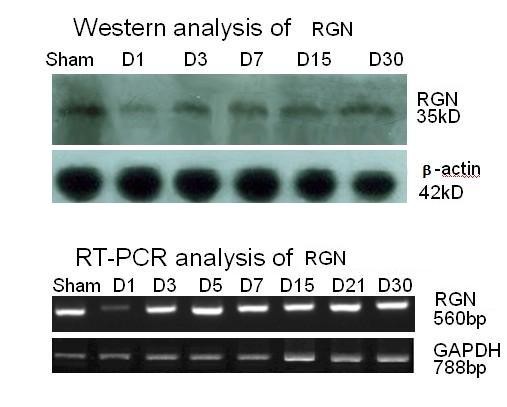
**Time course of the level of protein and mRNA of Rgn in regenerating mouse liver following 50% PH**. Liver samples from either sham-operated or 50% PH group recovered for different time period were analyzed by Western blot (upper) or RT-PCR (lower) for detecting the protein and mRNA of Rgn, respectively, as described in Methods. β-actin and GAPDH was probed in Western blot and RT-PCR, respectively, as a reference protein.

Regucalcin(Rgn), a Ca^2+ ^binding protein that plays an important role in cellular calcium homeostasis [15], has previously been identified as an up-regulated protein in response to 30% PH [16] but a slightly down-regulated protein following 70% PH [17]. Based on our proteomic analysis, the protein level of Rgn was reduced by 2.8 fold in the regenerating liver 24 h post 50% PH (Table 3). Using RT-PCR and Western Blotting, we further followed the dynamic changes of this protein over a time period of 30 days. The down-regulation of Rgn at both mRNA and protein levels was confirmed, and there was a rapid but partial recovery of Rgn at both levels over time (Fig. 3).

### Pathways regulating liver regeneration following 50% PH

**Figure 4:**
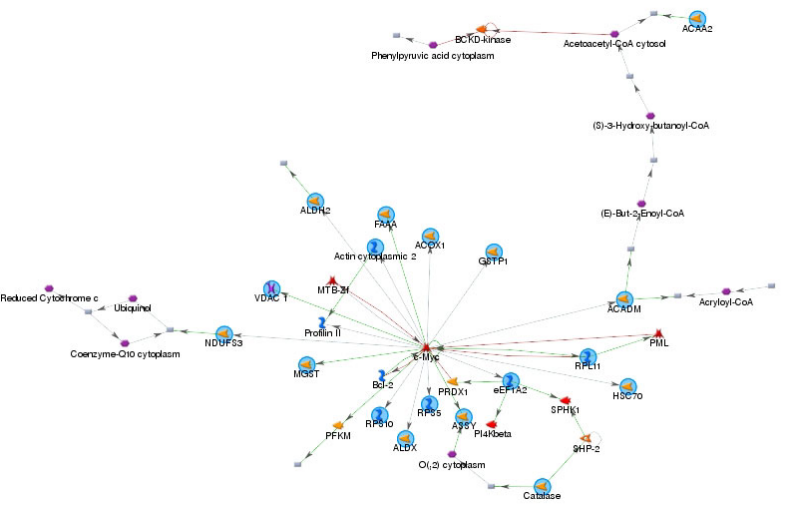
**Pathway analysis result showing 18 differentially expressed proteins following 50% PH can be sorted into the c-myc pathway**. There are 18 root nodes in the sub-network shown, of which ACAA2, ALDH2, ACOX1, GSTP1, ACADM, ASS1(ASSY) are up-regulated and VDAC1, MGST, RPL11, ALDX, RPS5 are "newly-induced" in response to 50% PH; ACTG1(Actin cytoplasmic 2), HSPA8(HSC70), CAT(catalase) are down-regulated and FAH(FAAA), NDUFS3, RPS10, eEF1A2 are below detection limit. These root nodes are connected to c-Myc directly or indirectly, and their expression levels or activities can be regulated by c-Myc, and some of them (such as RPL11) can regulate c-Myc activity in a feedback manner. Legend describing symbols in the network can be found in Additional file 1.

When all the 87 differentially-expressed proteins were pooled together for pathway analysis, 18 of them can be clustered into the same pathway and connected to each other in one way or another (Fig. 4 and Additional file 1). c-Myc situates at the center of this network. Among them, ACAA2, ALDH2, ACOX1, GSTP1, ACADM, ASS1(ASSY) were up-regulated and VDAC1, MGST, RPL11, ALDX, RPS5 were "newly-induced" in response to 50% PH; ACTG1(Actin cytoplasmic 2), HSPA8(HSC70), CAT(catalase) were down-regulated and FAH(FAAA), NDUFS3, RPS10, eEF1A2 were below detection limit.

## Discussion

Studies with hepatic resections have shown that within a certain range, the regenerative response is proportional to the amount of liver removed [18]. In most cases, removal of up to 75% of the liver was tolerated in patients with normal liver function pre-operatively [4], but greater than 75% to 80% resection is associated with a high mortality rate because of liver failure [3,4]. Therefore, to stimulate maximal liver regeneration yet maintain sufficient hepatic functionality, 70% (or 2/3) PH becomes the most commonly-used model to study the molecular mechanisms of rodent liver regeneration [19]. Previous results showed that many factors such as hepatocyte growth factor (HGF) [20], nuclear factor-kB (NF-kB) [21], tumor necrosis factor (TNF) [22] and interleukin (IL) 6 [23] might participate in the initiation of liver regeneration. In addition, serotonin, a neurotransmitter transported within the peripheral circulation by platelets, appears to be a co-mitogen that is essential for hepatic regeneration [24]. Based on the 70% PH model, a number of large-scale genomic [25-27] and proteomic [17,28-30] studies have been performed to map the complex dynamic changes of gene/protein expression during the process (in particularly the early phase) of liver regeneration, and various differentially regulated clusters of genes/proteins have been reported.

However, a lower percentage (e.g., 40-50%) of PH is more frequently conducted in partial liver transplantation [7] where hemiliver is removed from the donor. Moreover, resection must be more conservative in the presence of liver diseases (such as hepatic steatosis and cirrhosis), in patients after chemotherapy or in elderly patients [7]. Following removal of up to 50% of functioning liver, there was usually only a mild and short-lived increase in serum bilirubin and depression of serum proteins indicating sustained conservation of hepatocellular function [31]. In contrast to 70% PH, however, the molecular events of liver regeneration following 50% PH are much less understood and documented. We therefore sought to determine the global expression profile of proteins in the regenerating mouse liver following ~50% PH, and thereby gaining some insights into the hepatic regeneration mechanism(s) under this milder but clinically more relevant condition.

We grouped all the identified proteins into four categories that are deferentially expressed between mice subject to 50% PH and sham-operation. In group 1, 17 proteins were found to be up-regulated in 50% PH as opposed to the sham-operated group. Among them, 4 proteins have previously been associated with liver regeneration through proteomic studies: ALDH2 [17], CPS1 [17], HSPA9 [17], and CAR3 [30]. Firstly, mitochondrial aldehyde dehydrogenase 2 (ALDH2), the enzyme oxidizes a wide variety of aliphatic and aromatic aldehydes and converts them to acetic acid, which plays an important role in the clearance of ethanol-derived cytotoxic acetaldehyde [32], was found to be dramatically up-regulated by 14.3 fold in our study as compared to 2.8 fold increase in the study of Deng et al. [17] where regenerating rat livers were analyzed 4 h post 70% PH. Activation of ALDH2 correlates with reduced ischemic heart damage in rodent models most likely through reducing the formation of cytotoxic aldehydes [33]. Up-regulation of this enzyme at the protein level in liver cells was recently reported for rats exposed to heat stress [34]. Mitochondrial ALDH2 also plays a key role in bioconversion of nitroglycerin to its active vasodilator metabolite, and its oxidative inactivation by mitochondrial reactive oxygen species (ROS) underlies the development of nitrate and cross-tolerance [35]. Based on our pathway analysis (Fig. 4), ALDH2 is sorted into the c-Myc pathway which plays a critical role in regulating liver regeneration [36]. Secondly, HSPA9, which belongs to the heat shock protein 70 family, plays a role in the control of cell proliferation, and may also act as a chaperone [37,38], was found to be up-regulated by 3.6 fold, comparable to ~2 fold up-regulation reported by Deng et al. [17].

According to the Ingenuity Pathway Analysis conducted by Deng et al. [17], both ALDH2 and HSPA9 can be sorted into the Akt-NF-κB-p38MAPK signaling pathway, which is known to be essential for cell proliferation and liver regeneration [39,40]. Thirdly, another up-regulated protein found in both our study (by 6.7 fold) and in the report of Deng et al. [17](by 2.2 fold) is CPS1. It is primarily localized to liver mitochondria and plays a key role in the urea cycle. Patients with defects in the function or expression of CPS1 suffer from hyperammonemia [41]. It was found to be present in high concentrations in the plasma of septic humans, and thus to be considered as a biomarker for mitochondrial damage and depletion. This mitochondrial depletion is not due to cell death but is apparently related to the removal of damaged mitochondria by lysosomes (i.e., autophagy), followed by repletion of mitochondrial populations [42]. In Deng et al. study [17], CPS1 was sorted into the RB1-TP53-Myc pathway, which is known to be related to apoptosis, proliferation and cell-cycle progression [43-45]. Fourthly, CAR3 was found to be up-regulated by 2.4 fold in our study. The induced expression of this enzyme was found to be accompanied by increased cell proliferation and oxidative stress in mouse kidney [46]. Interestingly, CAR3 was found to be slightly down-regulated in regenerating mouse liver 1 to 3 days post 2/3 PH [30], and the cause for the discrepancy between the two studies remains unknown.

Besides the above 4 proteins which were also identified by other researchers, the augment of the remaining 13 proteins at the protein level has not yet been reported although some of them were found to be up-regulated at the mRNA level [47]. Among them, of particular note are ACOX1 (3.9 fold increase), the initial rate-limiting enzyme in the conversion of long-chain fatty acids to acyl-CoA thioesters, which donates electrons directly to molecular oxygen thereby producing hydrogen peroxide [48]. Also, this protein is sorted into the c-Myc pathway in our pathway analysis (Fig. 4). Elevation of ACOX enzyme activity (by ~3 fold) was reported for regenerating mouse liver 1 day after 70% PH, and was considered to result directly from transcriptional up-regulation by the transcription factor, peroxisome proliferator-activated receptor-α (PPARα) [49]. It is well established that numerous genes encoding peroxisomal, mitochondrial, and microsomal enzymes contain functional PPARα -responsive elements in their promoter regions, and these PPARα -responsive genes are largely involved in lipid metabolism, β-oxidation of fatty acids, and isoprenoid synthesis, processes required for cell proliferation [49]. Thus, identification of ACOX1 as an up-regulated protein in our system may indicate the global up-regulation of the PPARα-regulated pathways. However, the parallel induced expression of RPL11, a negative regulator of PPARα [50], may indicate a well-balanced control of this signaling pathway via feedback mechanism during liver regeneration. Since HP represents a major injury to the liver and to the whole body as well, it is expected to see altered expression of inflammatory and stress factors. Indeed, protein disulfide isomerase family which represents an injury response signal that can activate tissue factors to initiate blood coagulation [51] and to support extracellular matrix assembly [52], was up-regulated by 2 to 3.9 fold; in broad sense, HSPA9, as has been mentioned above, also belongs to stress responsive proteins.

In group 2, 33 proteins were detected only in 50% PH group but not the sham-operated group, and therefore are termed "newly-induced proteins". The failure of detecting those proteins in sham-operated group could be due either to true absence of expression of those proteins or to their very low quantity which is below the detection limit of our assay. In the latter case, those proteins are actually also up-regulated ones which are essentially the same as those classified into Group 1. About half (15 out of 33) of the "newly-induced proteins" in response to 50% PH belongs to cell regeneration related proteins, and among them, 5 are Histone (H_2_B) proteins, the post-translational modifications of which are known to be important for regulating gene transcription [53]. This finding is in good agreement with the one reported by Lim et al. [54] where the newly synthesis of the DNA and the mRNA of H_2_B was found to peak at 24 h and 36 h, respectively, after PH.

Keratin represents another major group of protein specifically detected in the regenerating mouse liver. Keratins are the largest subfamily of intermediate filaments consisting of >50 unique gene product members which include 37 epithelial and 17 hair keratin members in humans [55]. The hepatocytic keratin network is dense, particularly around bile canaliculi and at the cell periphery, and acts as cytoskeletal backbone to the functionally more dynamic and contractile actin microfilament system [56]. Keratins exhibit anti-oxidative and anti-apoptotic properties through sequestering oxidatively damaged proteins or apoptotic proteins, and are involved in cell protection [57]. Although there is evidence that keratin filaments can modulate hepatocyte mitotic progression [58] and liver regeneration [59], our present study showed for the first time the induced expression of keratins in regenerating livers. The rest of the newly-induced proteins are mainly those involved in carbohydrate, lipid synthesis, amino acid and nucleic acid metabolism. One of them, OAT, is a mitochondrial matrix enzyme that controls the L-ornithine level in tissues by catalyzing the transfer of the delta-amino group of L-ornithine to 2-oxoglutarate [60]. In agreement with our finding, an up-regulation of this protein was also reported by Hsieh et al. [30] where regenerating mouse livers following 2/3 PH were analyzed.

Group 3 and group 4 proteins are down-regulated or below detection limit with 50% PH treatment, respectively. Overall, the extent of down-regulation (as reflected by fold change or ratio) of proteins was not as dramatic as that of up-regulation, and was all below 4.2 fold reduction. Among those down-regulated proteins, the enzymes involved in ATP production and tubulin-like cytoskeleton proteins are particularly noteworthy. ATP5A1 and ATP5H which encodes α- and d- subunit of mitochondrial ATP synthase F1 and F0 complex, respectively, was down-regulated by 1.5-4.2 fold. Mitochondrial ATP synthase catalyzes ATP synthesis, using an electrochemical gradient of protons across the inner membrane during oxidative phosphorylation. The down-regulation of these ATP synthase subunits may lead to a decrease in cellular ATP production. Consistent with this is the finding that a number of key enzymes (such as SDHB, MDH2, NDUFS3) involved in citric acid cycle and the electron transport chain were also down-regulated or below detection limit, which could blunt the oxidative phosphorylation processes that are critical for ATP production. Indeed, Crumm et al. [61] reported that the ATP levels in remnant livers following both 30% and 70% PH decreased markedly and rapidly (to 48% of control by 30 seconds post-PH) and remained significantly lower than those in sham-operated controls for 24 to 48 hours. The reduction of ATP content in regenerating liver was not caused by increased energy demand, but by ATP release from the liver, and could generate early stress signals that contribute to the onset of liver regeneration. Our findings here provide important molecular mechanism for the phenomenon.

Tubulins are the main component forming the microtubule cytoskeleton in eukaryotic cells and play a key role in the regulation of the mitotic spindle for centrosome/chromosome movements in cell division and specific neuronal functions [62]. The expression level of tubulin subunits is relatively stable under most conditions. However, a strong down-regulation of β-tubulin mRNA was seen with hepatocytes treated with a nephro- and hepatotoxic mycotoxin [63]. We found a 1.4-2.5 fold decrease in α-tubulin protein level in regenerating liver, which is in contrast with the up-regulation of keratin as mentioned above. Interestingly, an opposing regulatory pattern of these two cytoskeletal components in liver tissues has also been seen in patients with hepatocellular carcinoma where β-tubulin protein level was up-regulated and keratin 8 down-regulated [64]. It remains to be investigated whether the opposing regulatory pattern of tubulin and keratin has any functional implications.

Remarkably, over 25 out of the 87 differentially-expressed proteins are located at the mitochondria, including the down-regulated ones mentioned above that are involved in ATP production. Thus, our study provides novel evidence for mitochondria as a pivotal organelle that is connected to liver regeneration. Recently, subproteomic analysis of proteins in isolated rat liver mitochondria after 70% PH revealed 25 differentially regulated proteins of which approximately half was identified in our current study [65]. However, in that study, only 3 proteins were found to be up-regulated while all the other 22 proteins (including ALDH, SUCLG2 which are up-regulated in our study) down-regulated. Since it has been reported that some proteins are released from mitochondria during liver regeneration after PH [66], it is conceivable that some of the up-regulated proteins detected in whole liver homogenate (such as ALDH2 in our study) may release from mitochondria into the cytosol, leaving their contents in isolated mitochondria even lower than those present in sham-operated control. Thus, subcellular fractionation and/or histochemistry/immunohistochemistry based studies will be helpful in determining the dynamic spatial and temporal changes of those identified mitochondrial proteins during the regeneration process.

## Conclusions

Taken together, compared to sham-operated group, we detected totally 87 differentially expressed proteins (with 50 up-regulated and 37 down-regulated ones) in the regenerating mouse livers following 50% PH, a condition that is clinically relevant to liver transplantation and conservative liver resection. Among them, over 25 are located at the mitochondria, and the majority is reported to be associated with liver regeneration for the first time. Consistent with the documented regulatory roles of c-Myc during the G_1 _phase of liver regeneration [67] and in the "priming" of hepatocytes [17,68], 18 of the identified differentially-expressed proteins are connected to c-Myc in a complex signaling network via multiple modes, and thus placing c-Myc as one of the master factors regulating liver regeneration under our experimental conditions. Importantly, the functional roles of some newly identified proteins in liver regeneration await further clarification, and whether they are differentially regulated specifically with 50% PH but not with other volume of PH is currently under investigation. Furthermore, in future, proteomic analysis-based time course studies will be useful to unravel the mechanisms that govern cessation of the regenerative process once the original liver mass has been restored.

## Methods

### Animal models

34 male C57/BL6 mice, with a mean weight of 20 g each, were purchased from Sichuan Academy of Medical Science Institute of Laboratory Animals. Animals were kept under standard conditions with a 12-hour day/night cycle and access to food and water *ad libitum*. Animal protocols were approved by the Ethics Committee of the First Affiliated Hospital of Zhejiang University before the commencement of the study.

These mice were divided into the no surgery control group (2), 50% PH group (28), and sham-operation group (4) by using a random number table. Before the operation, 3 liver samples were resected from the no surgery control group via the abdominal cavity. For the 50% PH group, approximately 45% -50% of the liver was resected via lobectomy from the lower-left region of the liver along the margin of the fourth bile duct. Two mice were sacrificed after operation at the following time points: Day 1, 3, 5, 7, 9, 11, 13, 15, 18, 21, 25, 30, and 36. Of the 3 liver tissue samples resected from each mouse, one was fixed with 4% paraformaldehyde and paraffin-embedded for biopsy, and the other two samples were stored at -80°C until samples from all time points are ready to be analyzed simultaneously. Two mice from the 50% PH group were dead after the operation.

### Extraction and purification of proteins

10 ml of T-PER^® ^Tissue Protein Extraction Reagent (Pierce, Rockford, IL USA), supplemented with 1 mM PMSF (Pierce, Rockford, IL USA) and 5 mM EDTA were prepared prior to use. The resected liver samples were washed in pre-chilled PBS and two samples were combined for each time point. The samples were grinded into a homogenous mixture via Sample Grinding Kit (Amersham Biosciences, Piscataway, NJ USA.); centrifuged at 10,000 × g for 15 min. The SDS-PAGE Sample Prep Kit (Pierce, Rockford, IL USA) was used for protein purification and the BCA™ Protein Assay Kit (Pierce, Rockford, IL USA) for protein quantification.

### Separation of proteins by SDS-PAGE

60 μg of each sample was separated on 10% SDS polyacrylamide gels. Gels were stained with Colloidal Coomassie Brilliant Blue G (Sigma, St Louis, MO USA) for 4 hours, and then were destained in a destaining solution (40% methanol, 10% acetic acid). Gels were scanned with ImageScanner II (Amersham Biosciences) and band intensities quantified by Volume Integration method using ImageQuant TL software (version 2005, Amersham Biosciences). The gel loading of 50% PH samples was confirmed to be between 100 ± 10% of that of the sham control in most cases, and was normalized in a few cases, by the total band intensity of each lane in the same gel.

### In-gel digestion and sample preparation

The entire lane was cut into 19 strips according to the visible bands. Each fragment was cut into 1~2 mm^3 ^gel pieces. In-gel reduction was performed with TCEP (Sigma, St Louis, MO USA) for 10 minutes at 60°C and carbamidomethylation was conducted with IAA (Sigma, St Louis, MO USA) for 1 hour at room temperature in the dark. The samples were dried via Speedvac (Labconco Centrivap Concentrator, Kansas City, MO USA). 15 μl of 25 mM NH_4_HCO_3 _buffer containing sequence grade trypsin (Promega, Madison, WI USA.) was added to each tube and each sample was allowed to rehydrate at room temperature for 15 minutes. Tryptic digestion was carried out overnight at 37°C. Peptides were extracted by washing the gel pieces three times with 50% acetonitrile and 5% formic acid. The supernatant was concentrated by a SpeedVac and was re-suspended in 30 μl of water with 0.1% formic acid for mass spectrometric analysis. All samples were stored at -20°C and were centrifuged at 15,000 × g for 5 minutes prior to mass spectrometric analysis.

### Protein identification and analysis

#### NanoUPLC conditions

Tryptic peptide separations were performed with a nanoACQUITY UPLC™ System (Waters Corporation, Milford, MA, USA), using a trapping column (Symmetry^® ^C18, 5 μm, 180 μm × 20 mm) (Waters Corporation, Milford, MA) and an analytical reversed-phase column (BEH130 C18, 1.7 μm, 75 μm × 150 mm) (Waters Corporation, Milford, MA), Samples were initially loaded and transferred with aqueous 0.1% formic acid solution to the trapping column at a flow rate of 15 μl/min for 1 min. Mobile phase A was water with 0.1% formic acid and B was 0.1% formic acid in acetonitrile. The peptide was separated with a gradient from 3% B to 40% B in 90 minutes, then to 90% B for 10 minutes, and held in 90% B for 10 minutes, at a flow rate of 300 nl/min. The column was re-equilibrated at initial conditions (3% B) for 30 minutes.

#### Q-Tof Premier mass spectrometer conditions

Mass spectrometry (MS) analysis of tryptic peptides was performed using Q-Tof premier™ mass spectrometer (Waters Corporation, Milford, MA, USA) with a nanoLockSpray™ source. External calibration was initially performed and the data subsequently post-acquisition lock mass corrected using Glu-fibrinopeptide (MW 785.8426), to ensure high mass accuracy. Data was acquired in the range from 50 Da to 1990 Da with the positive V-mode using alternating low collision energy (4 e V) (MS mode) and the course of the high energy was stepped from 15 to 40 e V (MSE mode). An integration time of 0.6 seconds was used for each scan. The reference, lock mass, channel was sampled every 30 seconds.

### Data processing and protein identification

**Figure 5:**
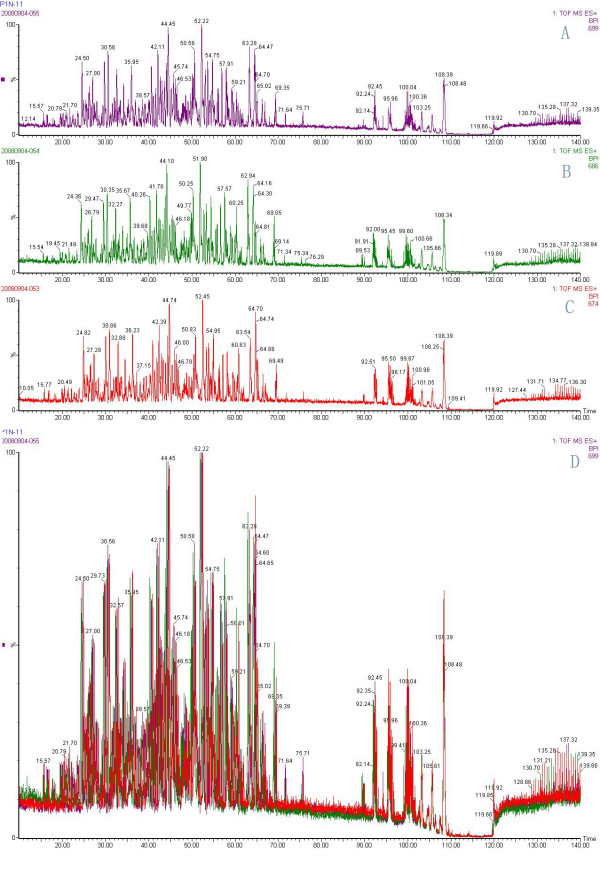
**Representative mass spectra of triplicate samples on nanoLC-Q-Tof Fig.5 (A-C) Individual mass spectrum of each of the triplicate samples shown in different colors. Fig.5 (D) An overlay of the mass spectrum of the triplicate samples**. The high degree of overlapping of most peaks present in the triplicate samples demonstrated good reproducibility of the method. The protein identification was conducted separately with the triplicate samples, and only an identification observed in at least two of the three replicates was taken to be valid. In those cases, at least two out of the three mass spectra exhibited high degree of overlapping as shown in this figure.

The continuum LC-MS data were processed and searched against the ipi.MOUSE.v3.40.fasta protein database http://www.ebi.ac.uk/IPI/IPImouse.html using ProteinLynx Global Server (PLGS) version 2.3, a fully automatic and integrated Mass-Informatics™ platform for quantitative and qualitative proteomics research[69]. Stringent search criteria were set so that each protein identification was collectively determined by at least 7 tryptic fragments and a matching peptide whose identification was determined by at least 3 tryptic fragments. Protein identification scores were calculated by the software and reported in the tables which provide a measure of confidence for the proteins identified. Since the LC-MS was performed in triplicate, the triplicate data were processed separately, and only an identification observed in at least two of the three replicates was taken to be valid (Fig. 5). The average MS signal of the three most intense tryptic peptides from each protein was taken as a measure for the relative quantity of that protein[70], and the ratio of this signal in 50% PH samples versus that in sham control sample was calculated and used to determine up or down-regulation of the specific protein upon 50% PH treatment. The gel equal loading validation/correction, and the triplicate MS identification, collectively ensured the authenticity of the results.

### Validation of the expression of selected proteins by Western blot analysis and RT-PCR

#### Western blot

Proteins were separated on 10% polyacrylamide gels and were transferred to PVDF membranes (Millipore, Bedford, MA, USA). The blots were incubated for 2 hours at room temperature in the TBST buffer (20 mM Tris-Cl, 140 mM NaCl, Ph 7.5, 0.05% Tween-20) containing 5% skim milk. Blots were incubated with the primary antibody (such as anti-RGN, diluted at 1:500, Santa Cruz Biotechnology, CA, USA; or anti-β-actin, 1: 2000, Sigma) overnight at 4°C. After being washed for three times in TBST, the blots were incubated with horseradish peroxidase-conjugated secondary antibody (diluted at 1:5000), (Pierce Biotechnology, Rockford, IL, USA) for 1 hour at room temperature, and developed with ECL reagents (Pierce Biotechnology, Rockford, IL, USA).

#### RT-PCR

Total RNA was isolated from liver tissues with TRIzol Reagent (Invitrogen). First-strand Cdna was generated from 1 μg of total RNA using an oligo (Dt) primer and the ImProm-II™ Reverse Transcription System (Promega). PCR was performed using Taq DNA polymerase (Takara). The primers used are as follows: 5'-GGAGGCTATGTTGCCACCATTGGAAC-3' and 5'-TTCCCTCCAAAGCAGCATGAAGTTGTTTTA-3' for RGN (560 bp); 5'-GTGAAGGTCGGTGTGAACGGAT-3' and 5'-GCATCCTGCTTCACCAC CTTCTT-3', for glyceraldehyde 3-phosphate dehydrogenase (GAPDH) (788 bp) [71]. The samples were incubated at 94°C for 2 min, and then amplified for 30 cycles of denaturation for 30 s at 94°C, annealing for 30 s at 60°C, and extension for 59 s at 72°C. The PCR products were analyzed by fractionation on a 1.2% agarose gel and visualized with ethidium bromide staining. Images were captured using a gel documentation system.

### Pathway analysis

Pathway network analysis of all the identified proteins was conducted using the Shortest Paths and the Analyze Network options of the data mining software MetaCore version 5.4 (GeneGo, St Joseph, MI) as described in[72]. The Shortest Paths option connects all given objects with the shortest possible path, no more than 3 steps. The network is built dynamically, connecting all objects via direct mechanistic interactions based on manually curated literature evidence. It can then be used for local functional enrichment of the network itself, to associate the interconnected cluster of metabolites and proteins with specific processes or diseases.

## Abbreviations

PH: partial hepatectomy; SLT: split-liver transplantation; PMSF: pheylmethylsulfonyl fluoride; EDTA: ethylene diamine tetraacetic acid; PBS: phosphate buffered saline; SDS-PAGE: sodium dodecyl ulphate polyacrylamide gel electropheresis; TCEP: trichloroethyl phosphate; IAA: iodoacetamide; nanoUPLC-Q-Tof: nanoultra performance liquid chromatrography-quadrupole-time of flight; PVDF: polyvinylidene difluoride; H&E: hematoxylin & eosin; Rgn: regucalcin; HGF: hepatocyte growth factor; NF-Kb: nuclear factor kappa-light-chain-enhancer of activated B cells; TNF: tumor necrosis factor; IL: interleukin; LC: liquid hromatography; MS: mass spectrometry.

## Competing interests

The authors declare that they have no competing interests.

## Authors' contributions

All authors participated in interpretation of the findings. LL and HC were responsible for the conception and design of the study. QZ made the animal models. WX, XJ, JYu, GS and YW performed the protein identification and analysis. JYu, JYa, QPan, JL and XP performed molecular biology experiments. HC and YW drafted the manuscript. LL modified and commented on the draft and all authors read and approved the final version of the paper. All authors confirm that the content has not been published elsewhere and does not duplicate their published work.

## Supplementary Material

Additional file 1 **Symbols used for pathway analysis.** The file is provided by Bioinformatics Center, Shanghai Institutes for Biological Sciences, Chinese Academy of Sciences, which decodes all the symbols used for pathway analysis in Fig. 4 Click here for file
