# Supplementary material for: Proteomic analysis of regenerating mouse liver following 50% partial hepatectomy
Source: Proteome Sci. 2009 Dec 29;7:48. doi: 10.1186/1477-5956-7-48 (PMC2813229; doi:10.1186/1477-5956-7-48)

Network Objects

Click on any object in the network to obtain class info

Enzymes

Generic enzyme

KINASE

- Generic kinase
- Protein kinase
- Lipid kinase

PHOSPHATASE

- Generic phosphatase
- Protein phosphatase
- Lipid phosphatase

PHOSPHOLIPASE

- Generic phospholipase

PROTEASE

- Generic protease
- Metalloprotease

GTPase

- G-alpha
- RAS - superfamily

Channels/Transporters

- Generic channel
- Ligand-gated ion channel
- Voltage-gated ion channel
- Transporter

Receptors

- Generic receptor
- GPCR
- Receptors with enzyme activity

Generic classes

- Receptor ligand
- Transcription factor
- Protein
- Cell membrane glycoprotein
- Compound
- Predicted metabolite or user’s structure
- Inorganic ion
- Reaction
- DNA
- RNA
- Generic binding protein

G protein adaptor/regulators

- G beta/gamma
- Regulators (GDI, GAP, GEF)

Groups of objects

A complex or a group

Proteins or compounds physically connected into a complex or related as a group

Logical association

Related proteins or compounds are connected into groups. To see the relations (logical associations) within a group, use «Expand group» function in the scroll-down right-button menu. Use «Collapse logical relations» function to close the group.

Group 1

Custom association

Group of collapsed objects chosen by user

Object highlighting

Nodes and root nodes

Found object

Object selected on the search pane

Manually selected node(s)

Object(s) selected by ctrl + click on it or by click + drag rectangle around it

Highlight by mouse over

Highlight upstream objects

When the mouse is over an object (node on a network), the closest interacting nodes are highlighted in CYAN if the direction of interaction is **from** the initial object

Highlight downstream objects

When the mouse is over an object (node on a network), the closest interacting nodes are highlighted in yellow if the direction of interaction is **towards** the initial object

Root nodes

Root node(s) for network expansion (building)

Object(s) from a user-specified uploaded list or from experiments

Initial object(s)

Object(s) chosen to build the pathways **from**

Intermediate object(s)

Object(s) situated along the pathway

Terminal object(s)

Object(s) the pathways terminate **on**

Possible combinations of three above marks (except the first one)

Expression data

Overexpressed gene(s)

Genes with higher conditional expression level compared to the experimental "control"

Underexpressed gene(s)

Genes with lower conditional expression level compare to the experimental "control"

Mixed-expressed gene(s)

Genes with conditional expression level statistically different from the experimental "control", with the "sign" of expression varying in different experiments

Other marks

Red circle

The links terminated due to a restriction of the number of steps in network expansion.
Network may be expanded from such nodes

Blue circle

The links terminated due to network truncation.
Network may be expanded from such nodes

Interactions between objects

Click on any hexagon in the networks for interaction annotation

Link legend

Positive effect

Negative effect

Unspecified effect

Technical link

Mechanisms

Physical interactions

Binding

B Protein or compound binds other protein or compound

C **Cleavage**
Cleavage of a protein at a specific site yielding distinctive peptide fragments.
Proteolytic cleavage can be carried out by both enzymes and compounds

CM **Covalent modifications**
(neddylation/deneddylation, sumoylation/desumoylation, ubiquitination/deubiquitination and etc.) Protein activity regulation by covalent binding of a small chemical group to the aminoacids of an active site.

+P **Phosphorylation**
Protein activity regulation by an addition of a phosphate group

-P **Dephosphorylation**
Protein activity regulation by a removal of a phosphate group

T **Transformation**
Protein activity regulation by binding & hydrolysis of GTP

Tn **Transport**
Transport of a protein or a compound between organelles

Z **Catalysis**
Catalysis of an enzymatic reaction

Tr **Transcription regulation**
Physical binding of a transcription factor to target gene’s promoter

M **MicroRNA binding**
Regulation of gene expression by binding of microRNA to target mRNA

Functional interactions

IE **Influence on expression**
Protein’s or compound’s action results in changing the expression level of target gene(s)

Cn **Competition**
Protein activity regulation by competition at the substrate binding site

? **Unspecified interactions**
Mechanism is unknown or/and effect is indirect

PE **Drug-Drug interactions. Pharmacological effect**
Drugs change pharmacological effects of other drugs, for instance by competing for drug metabolism enzymes or organic transporters

TE **Drug-Drug interactions. Toxic effect**
Drugs change toxic effects of other drugs, for instance by competing for drug metabolism enzymes or organic transporters

Logical relations

GR **Group relation**
Object belongs to a generic group of related objects

CS **Complex subunit**
Protein is a subunit of a protein complex

SR **Similarity relation**
Chemically similar compounds with chosen Tanimoto similarity score

Connectors

Connectors

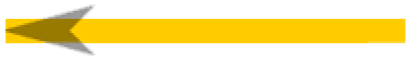
Incoming interaction
When the mouse is over an object, yellow link indicates direction **to** the object

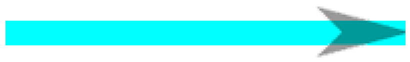
Outgoing interaction
Cyan link indicates direction **from** the object

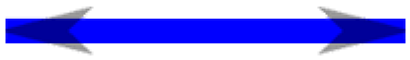
Bidirectional interaction
Blue link indicates **BI-DIRECTIONAL** interaction

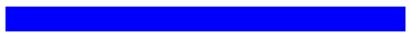
Non-directional link
Blue link also indicates an interaction for which the direction is not specified

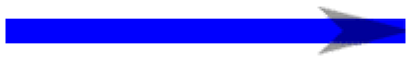
Traced link
The link is always highlighted in blue if both linked objects are selected in "Trace" mode

Interactions from custom list

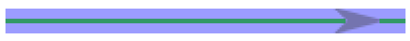

Interaction is in the network

Interaction is represented by a thin solid line and is highlighted in blue

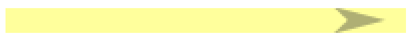

Interaction is in the base, but not in network

Interaction is highlighted in yellow

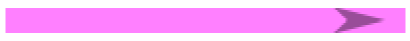

Interaction is not present in the base

Interaction is highlighted in magenta

Canonical pathways

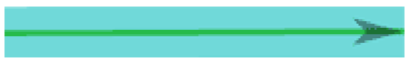

Canonical pathways

The link is highlighted in thick cyan line

Custom marked links (user's choice)

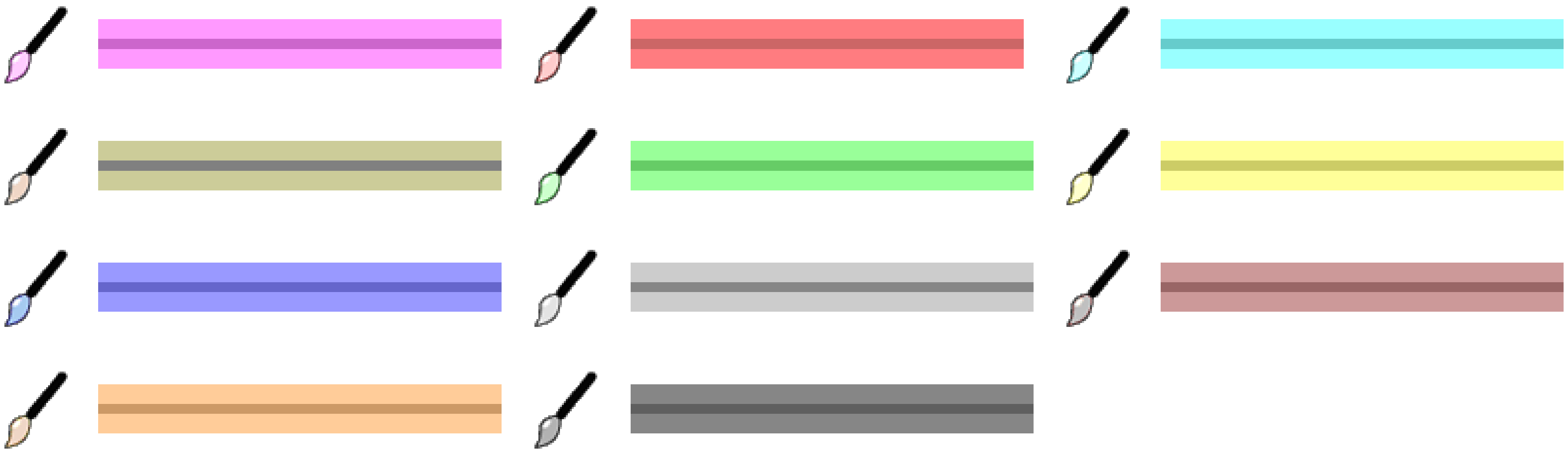

Supplement: Additional file 1 — Symbols used for pathway analysis. The file is provided by Bioinformatics Center, Shanghai Institutes for Biological Sciences, Chinese Academy of Sciences, which decodes all the symbols used for pathway analysis in Fig. 4 [file 1477-5956-7-48-S1.pdf]
